# A multi-analytical evaluation of the depositional pattern on open-air rock art panels at “Abrigo del Lince” (Badajoz, Spain)

**DOI:** 10.1007/s11356-022-23589-2

**Published:** 2022-11-07

**Authors:** Maria Nicoli, Negar Eftekhari, Carmela Vaccaro, Hipólito Collado Giraldo, Sara Garcês, Hugo Gomes, Virginia Lattao, Pierluigi Rosina

**Affiliations:** 1 Department of Architecture, University of Ferrara, Ferrara, Italy; 2 Department of Physics and Earth Science, University of Ferrara, Ferrara, Italy; 3 Department of Environmental and Prevention Sciences, University of Ferrara, Ferrara, Italy; 4 Polytechnic Institute of Tomar, Tomar, Portugal; 5 Geosciences Centre, University of Coimbra – (u. ID73-FCT), Coimbra, Portugal; 6 Department of Earth Sciences and Geosciences Center, Faculty of Sciences and Technology, University of Coimbra (Polo II), Coimbra, Portugal

**Keywords:** Schematic rock art, Badajoz, Granite, Pigment analysis, SEM–EDS elemental mapping, Weathering, Biofilm, Patina

## Abstract

Microscopic observation correlated with chemical–mineralogical characterization was performed on pigment samples from “Abrigo del Lince” rock art site (V-IV millennium BC), in order to provide contributions to the study of prehistoric schematic art on granite in the province of Badajoz (Spain). The research objectives include the understanding of technological and cultural aspects, as well as of conservation and deterioration issues related to the pictographs. The multi-analytical approach encompasses the integration of microscopic observation, SEM–EDS analysis, micro-Raman spectroscopy, and ATR-FTIR and allowed to achieve a multispectral overview of the samples and to describe their varied composition and the alteration pattern which connects them. The main phases overlying the granitic bedrock and involved in this sequence are as follows: hematite, whewellite, and gypsum. While hematite could be stratigraphically considered the most ancient layer and assigned to the use of red ochre as a pigment, whewellite and gypsum are the main constituent of the alteration layer which forms a patina over the pictographs, due to weathering processes. Finally, the role of biofilms in rock art conservation is discussed, suggesting that, especially for what concern thin and homogenous layers of oxalates, their presence should not be necessarily considered an issue.

## Introduction

The research of schematic art paintings in the province of Badajoz began at the beginning of the twentieth century. Since the first explorations, Extremadura experienced a considerable increase in the number of known rock art sites, which was accompanied by the growing production of specialized publications during almost a century of research (Domínguez García et al. 2010, 2013). The best-known works on twentieth-century schematic art in Spain were the works of Henri Breuil and Pilar Acosta. Henri Breuil devoted a lot of work to the discovery and documentation of sites specifically in the Guadiana River basin (Acosta Martínez 1968; Breuil 1933). Among the various works presented in recent years dedicated to the study of schematic painted rock art in the Extremadura area (Collado Giraldo 2009, 2007; Collado Giraldo and García 2017; Collado Giraldo et al. 2014, 2009; Collado Giraldo and García 2005; Collado Giraldo and García Arranz 2009; Collado Giraldo and García 2005; Domínguez García et al. 2013, 2010), there are some references to sites whose bedrock is granite (Collado Giraldo and García Arranz 2009).

The phenomenon of rock art on granite in the Extremadura community presents markedly differentiated implantation between the various provinces in the area. It is a reality much better known and documented in the region of Cáceres, where most of the sites known so far are located, than in that of Badajoz. In the province of Badajoz, the existence of painted granite shelters is still scarce. Obviously, even considering that the representation of granitic areas is somewhat greater in the province of Cáceres, the difference is not as marked as that shown by the current territorial distribution of sites with rock art on granite. This fact leads us to think that this reality is distorted by the lack of systematic research. This research aims to contribute to the development of studies about the distribution of rock art on granite support in the Badajoz province.

Moreover, the study of rock art from the archaeometric point of view can provide contributions in the understanding of technological and cultural aspects, as well as of those related to the conservation and deterioration of pictographs (Gallinaro and Zerboni 2021; Gheco et al. 2020;). This approach is currently becoming a standard in archeology and rock art studies, both for the physicochemical characterization of pigments (Iriarte et al. 2009) and for the detection of alteration products, such as oxalates, which presence is remarkable in the context of rock art preservation and datation (Ruiz et al. 2012; Sanjurjo-Sánchez et al. 2012; Green et al. 2017; Pozo-Antonio et al. 2017; Rousaki et al. 2018). This work aims to examine micro-stratigraphic relationship between rock, pigment, and alteration phases, using a multi-analytical approach. Furthermore, it aims to improve the knowledge and understanding of the materiality of rock art evidence and of its conservation (Sepúlveda 2021). Among the most valuable and effective techniques for the study of rocky substrate, pigments and patinas are SEM–EDS, micro-Raman, and FTIR spectroscopy, which enable to characterize them chemically and morphologically (Kurniawan et al. 2019; Chalmin et al. 2017). In the framework of micro-archeological approach, microscopic observations are particularly important in order to support more sophisticated investigation techniques and to highlight taphonomic processes occurring on rock art pictographs. In this study, the acquisition and comparison of multispectral microscopic views of the samples played a key role in order to collect a comprehensive data set and formulate a consistent interpretation to address the above questions.

## Geoarchaeological context

**Fig. 1:**
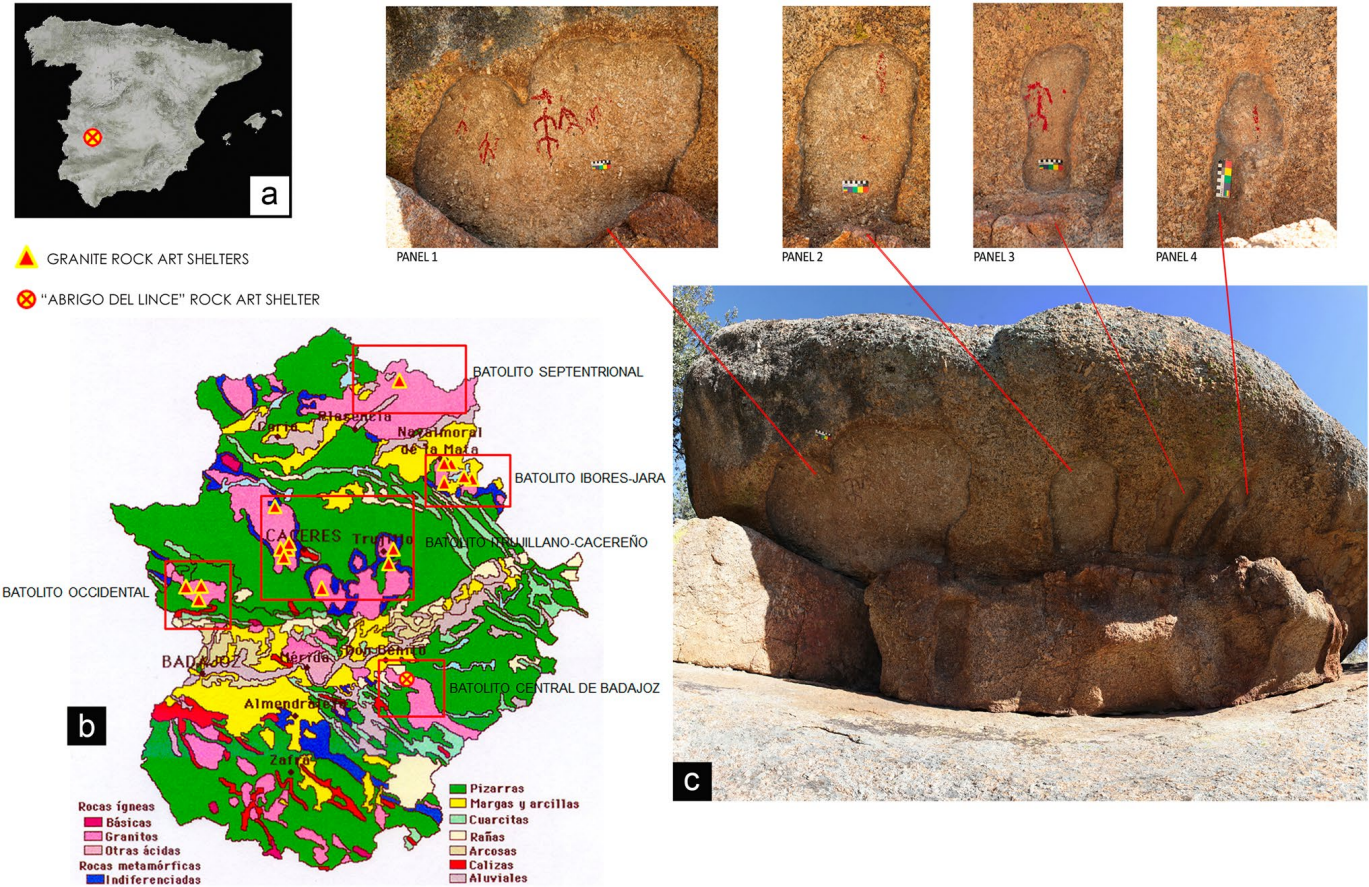
**a** Map of the Iberian Peninsula with the localization of “Abrigo del Lince” rock art site; **b** enlarged view of Extremadura region with the location of “Abrigo del Lince” rock art site and of other rock art shelter on granite in the same region; **c** general view of the shelter with the indication and detail of each painted panel on the rock stack

In Badajoz, the “*Abrigo del Lince*” shelter is known as the only rock art site whose bedrock is granite. This new site with rock art is located south of the town of Don Benito, in the natural area called “*La Serrezuela*”. The area is located from a geological point of view on the southern edge of the Central Iberian Zone corresponding to the central part of the tertiary basin of the western Guadiana. It is part of the physiographic region of the *Vegas Altas* del Guadiana, located in the northern central area of the province of Badajoz (Fig. 1a and b). It is a territory characterized by gentle hilly areas around 300 m altitudes, which is physiographically bounded to the north by the Guadiana basin; to the west by the *Sierra de la Ortiga*, where the main elevations are located, and to the east by the *Sierra de Magacela*. Granite is the main feature of the landscape, emerging in the form of rocky outcrops based on massive, rounded bodies of metric to decametric dimensions, where the piles of large blocks are characteristic. All this landscape is dotted by a very anthropized vegetation, with the presence of sparse and scattered holm oaks and a reduced cover of scrubland, mainly rockrose, due to the intensity of the agricultural exploitation, to which the area has historically been subjected. The *Lince* shelter is located on a hill that dominates the surroundings, although with a preferential orientation towards the southern area. The site is configured as a large pile of granite boulders, whose upper block generates in its lower part a wide visor. Under the protection of that visor, the paintings were represented, taking advantage of a series of natural hollowed-out hollows, which individualize each of the four documented panels. At the *Lince* shelter, eleven figures have been identified, which represent human figures and lines (Fig. 1c). There are no direct dates for this group of schematic rock art. Stylistically, these depictions could be dated to the Neolithic Age (V-IV millennium BC). The station measures 8.20 m in length by 3.10 m in maximum height, to the ceiling from the current ground level through the access area and field observation reveals that some spots of the site are subjected to bio-colonization.

## Materials and methods

### Sampling

**Fig. 2:**
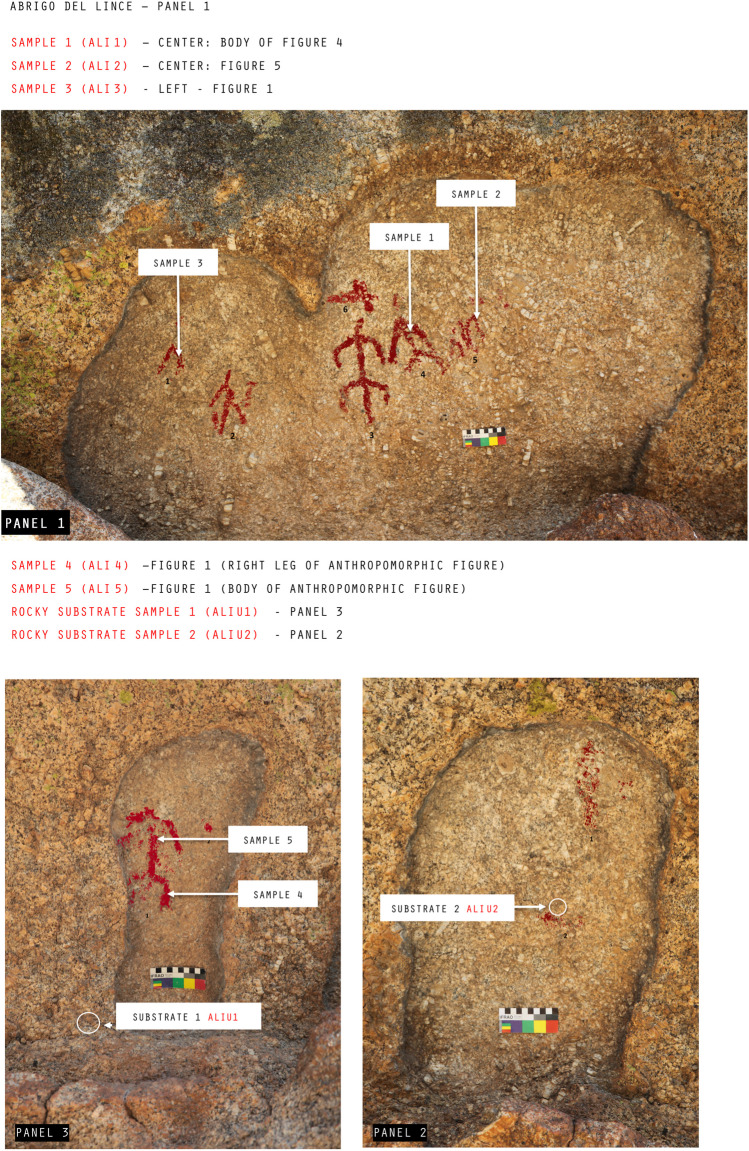
Detail of panels 1, 2, and 3 with anthropomorphic pictographs. Sample areas are indicated for each panel

From “*Abrigo del Lince*” shelter, five samples of pigments and two samples of the bedrock were selected, sampling location are shown in Fig. 2. In panel 1, three pictographs have been sampled: two human figures and a line figure. Two more samples have been taken from a human figure on panel 3. The choice of specific sites for sampling is related to pictographic interest, pigment availability and minor risk of damage. All samples were of red pigment and they were collected from strategic figurative motifs in order to encompass several types of motives. This authorized sampling strategy was made in compliance with national and international guidance on sampling, to assure that the integrity of the imagery was not compromised. Where possible, sample collection was undertaken using ethical extraction techniques followed by European Standard concerning sampling for investigation of cultural heritage  (EN 16085:^2012^). Each sample, weighing between 10 and 100 mg, was extracted in areas of the panel where the pigment was observed or in areas where minute fractures and niches were present. Normally, fractures and niches are considered from an ethical point of view the most preferred places to extract pigment, because it is a way to reduce the impact of sampling from a rock art surface. Each sample was obtained using a sterilized tungsten scalpel, and they consisted in small fragments of rock bearing a fraction of the painted figure. Samples were inserted into 0,5 ml microcentrifuge tubes.

Two samples from the undecorated wall blank (bedrock) were also taken to discriminate which mineral elements belong to the decorated part of the wall and what minerals formed the bedrock. A full resume of the samples considered in the present work is listed in Table 1.

**Table 1:** Sample description and summary of analysis results

Sample ID	Sampling location	Sample description	SEM–EDS	ATR-FTIR			Micro-Raman		
				Patina	Pigment	Substrate	Patina	Pigment	Substrate
ALI1	Panel 1: Fig. 4, body of the anthropomorphic figure	Red pigment	Fe, Si, Al, Ca, K, P, Mg, Na, S, Ti	Whewellite; carbonate	Hematite	Silicates; feldspar	Gypsum	Hematite	
ALI2	Panel 1: Fig. 5, red line figure	Red pigment	Fe, Si, Al, Ca, K, P, Mg, S					Hematite	Anorthoclase
ALI3	Panel 1: Fig. 1, anthropomorphic figure	Red pigment	Si, Al, Ca, Na, Fe, P, K, Mg, S, Ti	Carbonate; whewellite		Feldspar; silicates		Hematite	Albite
ALI4	Panel 3: Fig. 1, right leg of the anthropomorphic figure	Red pigment	Ca, Si, Al, Fe, K, Mg, S, P, Na, Ti, Cl	Whewellite; calcium phosphate		Feldspar; quartz	Gypsum	Hematite	Quartz
ALI5	Panel 3: Fig. 1, body of the anthropomorphic figure	Red pigment	Ca, Si, Fe, Al, S, K, Mg, Na, Cl	Whewellite; carbonate	Iron oxide	Feldspar; alumino-silicates; quartz	Whewellite; gypsum	Hematite	
ALISU1	Panel 3	Rocky substrate	Si, Al, Fe, K, Mg, Ca, Ti, Na, P, S	Feldspar, quartz, iron oxide					
ALISU2	Panel 2	Rocky substrate	C, O, P, Mg, Ca, S, Fe, K, Si, Mn, Al	Feldspar, whewellite, quartz, iron oxide, metal oxides			Quartz, biotite, orthoclase, whewellite, gypsum, calcium phosphate		

### Analytical methods

#### Microscopic observations

A stereomicroscope SZ6745TR equipped with a MOTICAM 2500 5.0 M pixel webcam was used to carry out microscopic study of samples, in order to steer subsequent analyses. Micro-Raman spectroscopy and SEM–EDS technique, in fact, were used to study samples surfaces. In addition, when dealing with cultural heritage, the dimensions of samples must be very small due to preservation purposes. For these reasons, microscopic observations are fundamental in order to study samples surfaces, identifying areas with significative concentration of pigment and locating them within a reference system, which can be used during further analysis. Images were captured using Motic Images Plus 2.0 ML software.

#### SEM–EDS

A ZEISS EVO MA 15 scanning electron microscope (SEM), coupled with an energy-dispersive X-ray spectroscopy (EDS) system (Oxford Instruments) and equipped with a silicon drift detector (SDD), a LaB6 filament as an electron source, and cobalt as a calibration standard, was employed for microstructural characterization and to determine the chemical compositions of samples. AZtec 3.3 software was used to collect and process SEM–EDS data. Small rock fragments and pigments were studied at 20 kV and an 8.5 mm working distance under variable pressure (VP) conditions. Images were captured using both back scattered electrons detector (BSD) and secondary electrons detector (SED). In order to preserve samples for future analyses, they were not coated and they were observed in variable pressure conditions. Moreover, SEM–EDS chemical mapping technique was applied to investigate elemental distribution within samples and to differentiate between rock, pigments, and patinas. Thus, for each pigment sample, micrographs, elemental mappings, and EDS spectra were acquired.

#### ATR-FTIR

Fourier transform infrared (ATR-FTIR) spectra of samples were collected using a Bruker Alpha FT-IR, Opus 7.5 software, spectrometer employing an ATR (attenuated total reflection) sampling device. The ATR-FTIR spectrometer was equipped with a globar source, a KBr beam splitter, and a Deuterated Lanthanum α Alanine-doped TriGlycine Sulfate (DLaTGS) detector in room temperature. Diamond, single-reflection ATR cell was used. Spectra were recorded over the spectral range of 400–4000 cm^−1^ at a 4 cm^−1^ resolution, 24 scans. The attribution of the detected phases was performed using bibliographic references for comparison and the RRUFF database (https://rruff.info/).

#### Micro-Raman spectroscopy

A LabRam HR800 micro-Raman from Horiba Scientific, equipped with an air-cooled CCD detector at − 70 °C, an Olympus BXFM microscope (objective 10 × and 50 ×), and a 600 groove/mm grating, was used to collect the Raman scattering signals of mineral phases present on the samples. The excitation source was a He–Ne laser (632.8 nm line) with a maximum laser power of 17 mW and the spectrometer was calibrated with silicon at 520 cm^−1^. Spectra acquisition and treatment were performed using HORIBA Scientific’s LabSpec 6 Spectroscopy Suite Software. Identification of the mineral phases and peaks attribution was done referring to BIO-RAD spectral database, using KnowItAll spectroscopy software.

## Results

Table 1 shows the main results of the multi-technique analytical approach carried out on the samples.

### ALI1

**Fig. 3:**
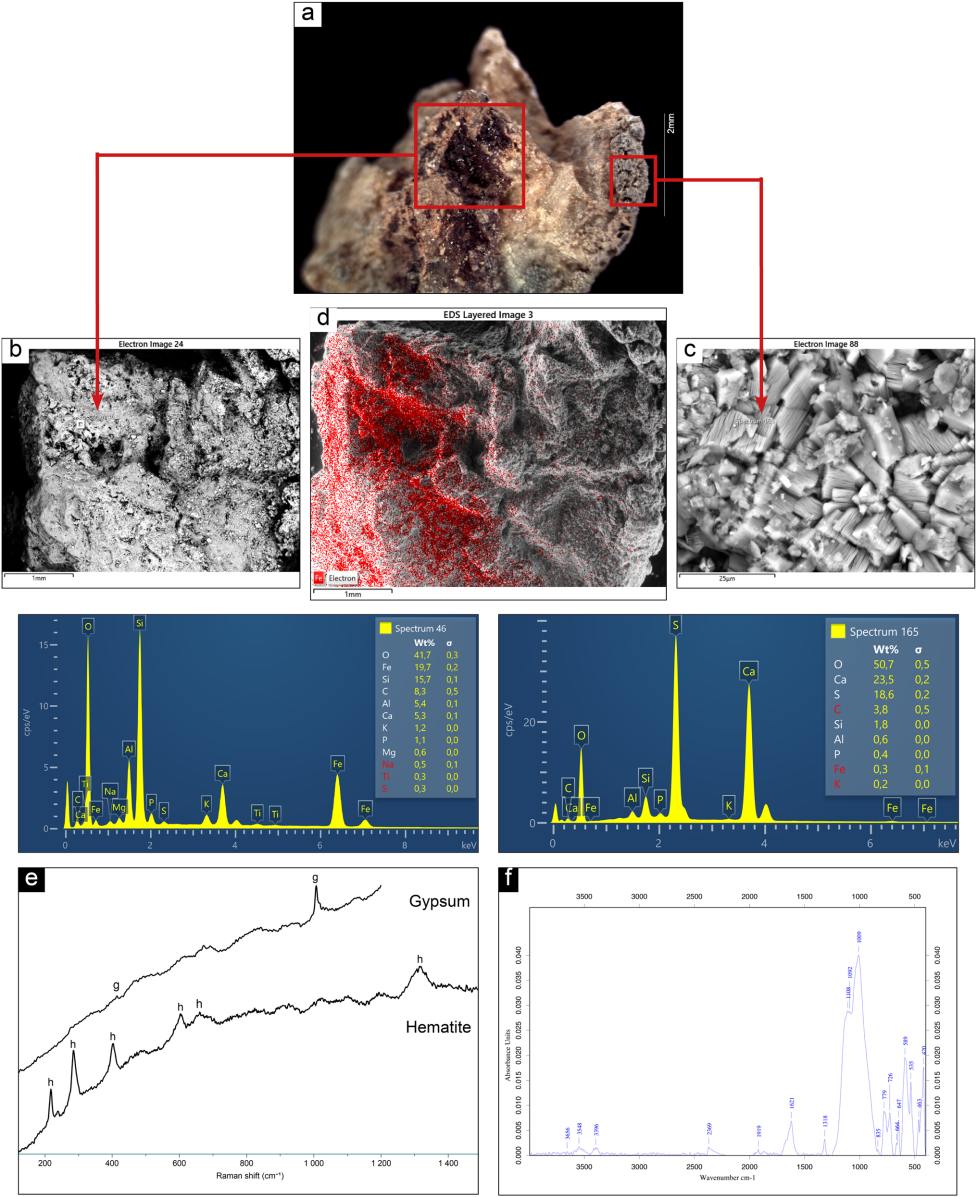
Summary of the analytical results obtained on sample ALI1: **a** microscopic view and indication of investigation areas; **b**–**c** SEM micrograph (BSE) of those areas and related EDS spectra; **d** SEM–EDS chemical mapping of Fe; **e** mineralogical phases detected by micro-Raman spectroscopy; **f** ATR-FTIR spectrum

Microscopic observations of the pigment sampled from panel 1 showed a deep red area over a rocky substrate (Fig. 3a). The first set of results from ALI1 refers to this reddish spot. The same area was observed using SEM–EDS technique, both in back scattered (BSE) and secondary electron mode. A small area, corresponding to a bright spot in BSE mode, was analyzed using EDS microprobe, in order to determine its semiquantitative chemical composition, which resulted in a compound enriched in Fe, besides other elements such as Si and Ca (Fig. 3b). In order to assess the distribution of Fe on the sample, a chemical mapping was performed. Fe distribution overlaps coherently with the reddish spots visible on the sample (Fig. 3d). Raman analyses were performed on the same areas to characterize the mineralogical phases within the reddish compound and hematite was detected (peaks at 217, 284, 401, 603, 660, 1315 cm^−1^). This result allows to relate the presence of Fe to the use of red ochre as pigment source. Gypsum as well was detected by means of μ-Raman spectroscopy (peaks at 413 and 1007 cm^−1^). Consistently, on the same sample, but in a different area, SEM–EDS analyses revealed high percentages of Ca and S and the clear morphology of gypsum crystals (Fig. 3c). The analyses on sample ALI1, therefore, showed that, while Ca seems to have a homogeneous distribution over the sample, the presence of sulfur appears to be restricted to a specific area, which does not match Fe distribution. This fact may suggest that gypsum is a secondary formation and not a pigment constituent. FTIR spectroscopy showed the presence of felspars and silicates pertaining to the granitic substrate, as well as the signal of whewellite (calcium oxalate monohydrate) and carbonate, accordingly with the observations made by SEM–EDS. The results of the analyses performed on sample ALI1 are summarized in Fig. 3.

### ALI2

**Fig. 4:**
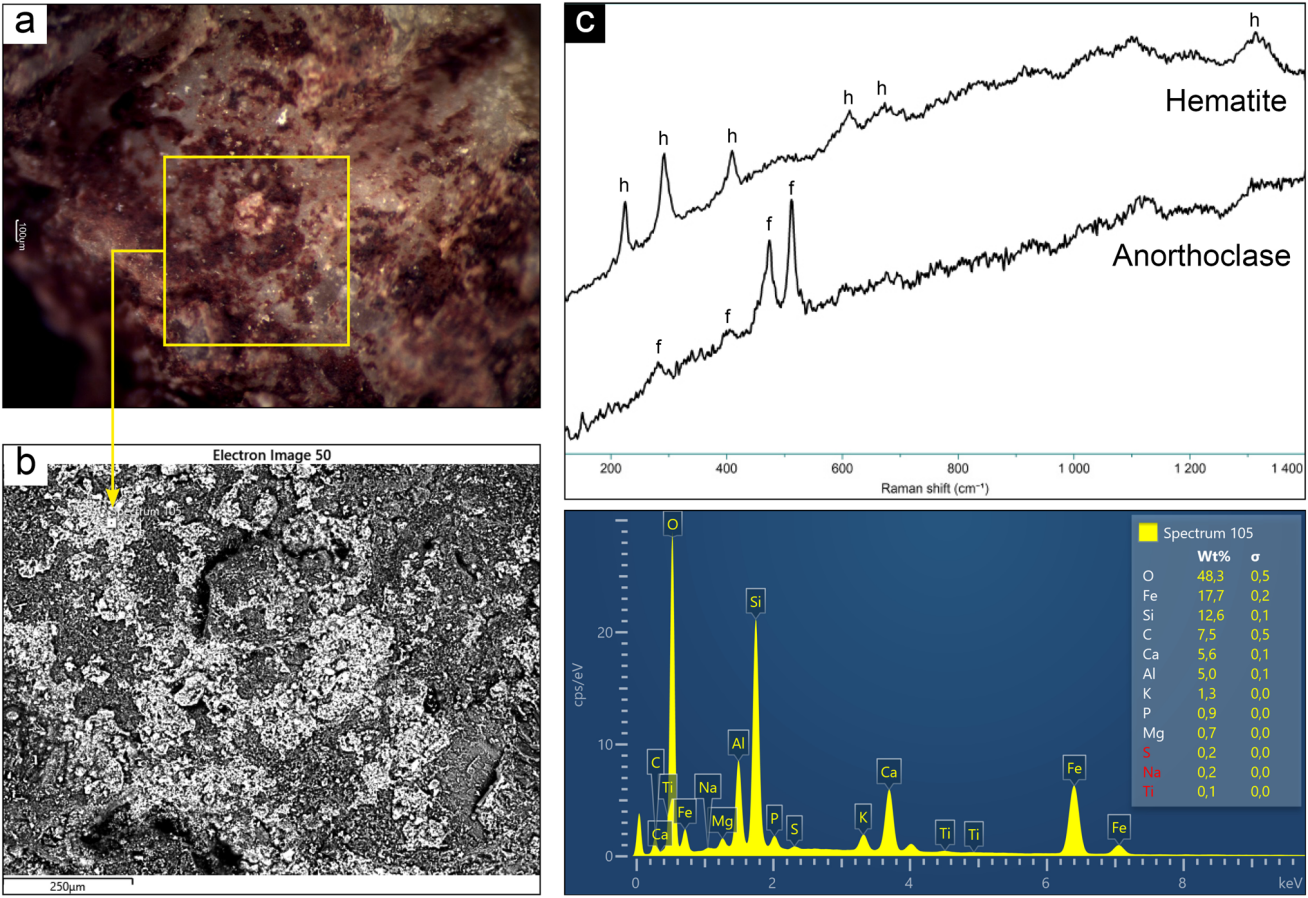
Summary of the analytical results obtained on sample ALI2: **a** Microscopic view and indication of investigation area; **b** SEM micrograph (BSE) of the area and related EDS spectra; **c** mineralogical phases of hematite and feldspar (anorthoclase) detected by micro-Raman spectroscopy

On sample 2, from the same panel 1, SEM observations in back scattered mode highlighted a neat distinction between bright and dark areas, suggesting the presence of two different chemical compounds. The distribution of bright areas seems to match the pattern of red spots observed on the sample through microscopic observations (Fig. 4a). SEM–EDS analyses carried out on those areas showed the presence of Fe in high percentages (Fig. 4b). Finally, the comprehensive chemical mapping of a wider area allowed to observe the spatial distribution of the Fe enriched compound onto the sample and suggested that it could be a secondary anthropic contribution. The result of Raman analysis seems to support this hypothesis as it provided the peaks of hematite at 223, 291, 408, 611, 672, 1312 cm^−1^ in the reddish areas (Fig. 4c). Hematite is a mineralogical phase typically found in red ochre used as pigments in prehistoric and historic times. Moreover, Raman inspection detected the phase of feldspar (anorthoclase) pertaining to the substrate, with the characteristic peaks at 281, 403, 473, 511 cm^−1^. No further information could be collected from this sample by FTIR spectroscopy.

### ALI3

**Fig. 5:**
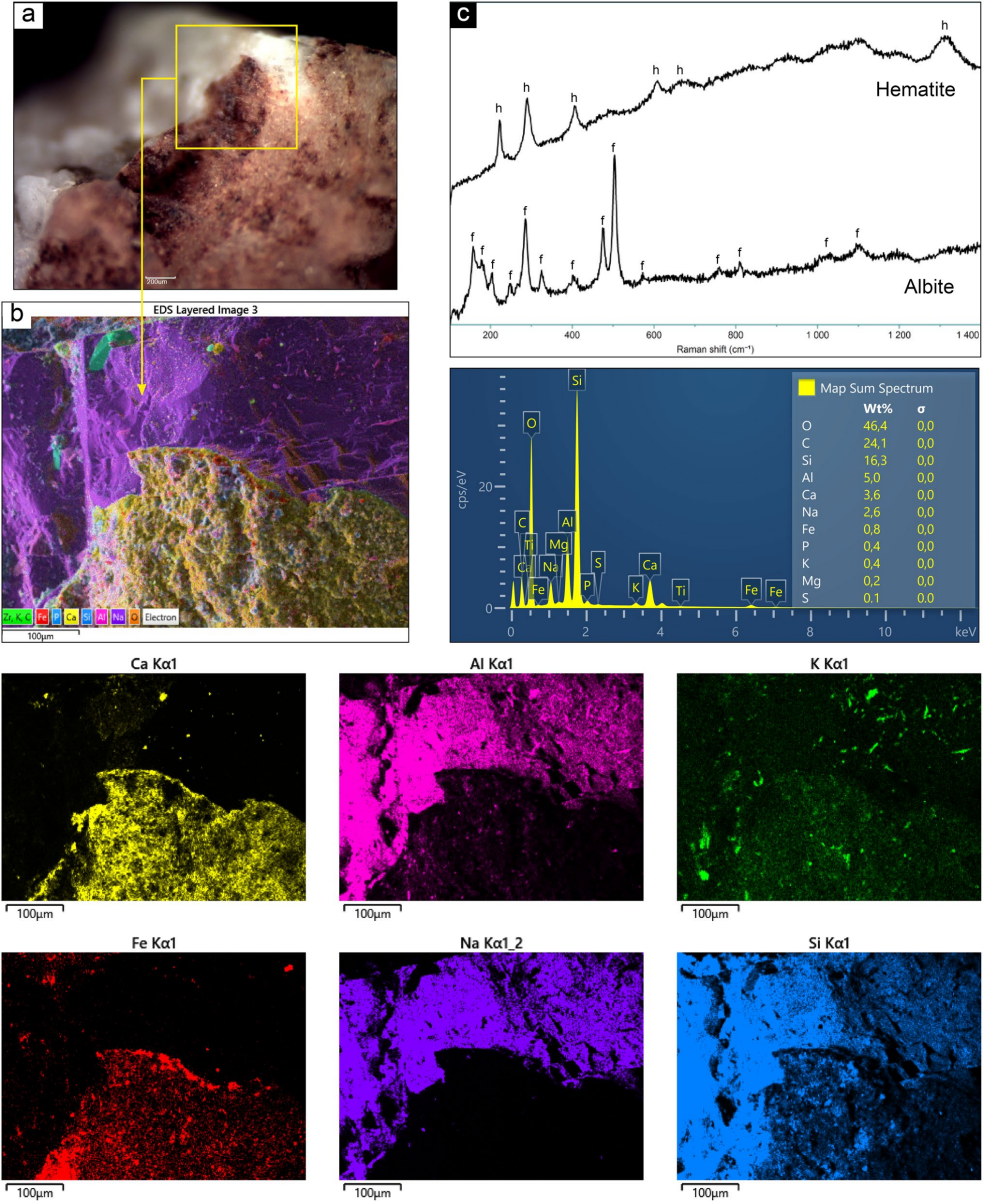
Summary of the analytical results obtained on sample ALI3: **a** Microscopic view and indication of investigation area; **b** SEM–EDS chemical mapping over the pigment area, showing the distribution of Ca, Al, K, Fe, Na, and Si, together with map spectrum; **c** mineralogical phases of hematite and feldspar (albite) detected by micro-Raman spectroscopy

The semi-quantitative chemical analyses, performed by means of SEM–EDS microprobe, did not show high percentages of Fe over sample 3. However, the chemical mapping performed later brought out a peculiar distribution of this element, compatible with the one of the areas with greater concentration of red pigment, observed through the microscope (Fig. 5a). The chemical map also presented a strong differentiation between elements reasonably pertaining to the rock substrate (Al, Na, Si) and those attributable to the superficial layers (Fe and Ca) (Fig. 5b). On the base of those data and observations, the hypothesis is that the microstratigraphy over the rock is indeed comprised of two layers: a lower one enriched in Fe, which is covered by a subsequent layer containing Ca. In some areas, for example where the specimen has been detached from the bedrock, the stratum underneath comes up. Cross-sections should be performed in order to assess that and differentiate more clearly between the two layers. However, the materials sampled for this study did not allow this further investigation, due to their small size and brittleness. The patina over the sample was analyzed through Raman spectroscopy, in order to identify the phases that comprise it and hematite was detected (peaks at 222, 240, 290, 408, 609, 670, 1313 cm^−1^), which is coherent with the reddish color of the layer and the use of red ochre as a pigment. No mineralogical phases attributable to the presence of calcium oxalates could be detected by micro-Raman technique (as the sample gave a strong fluorescence signal). Nevertheless, Raman spectra displayed the bands of albite (peaks at 158, 178, 204, 246, 285, 324, 402, 475, 502, 570, 758, 811, 1028, 1099 cm^−1^), attributable to the granitic substrate, characterized by feldspar and plagioclase (Fig. 5c). FTIR spectroscopy, instead, highlighted the presence of whewellite and carbonate, besides the strong contribution of feldspars and silicates pertaining to the substrate.

### ALI4

**Fig. 6:**
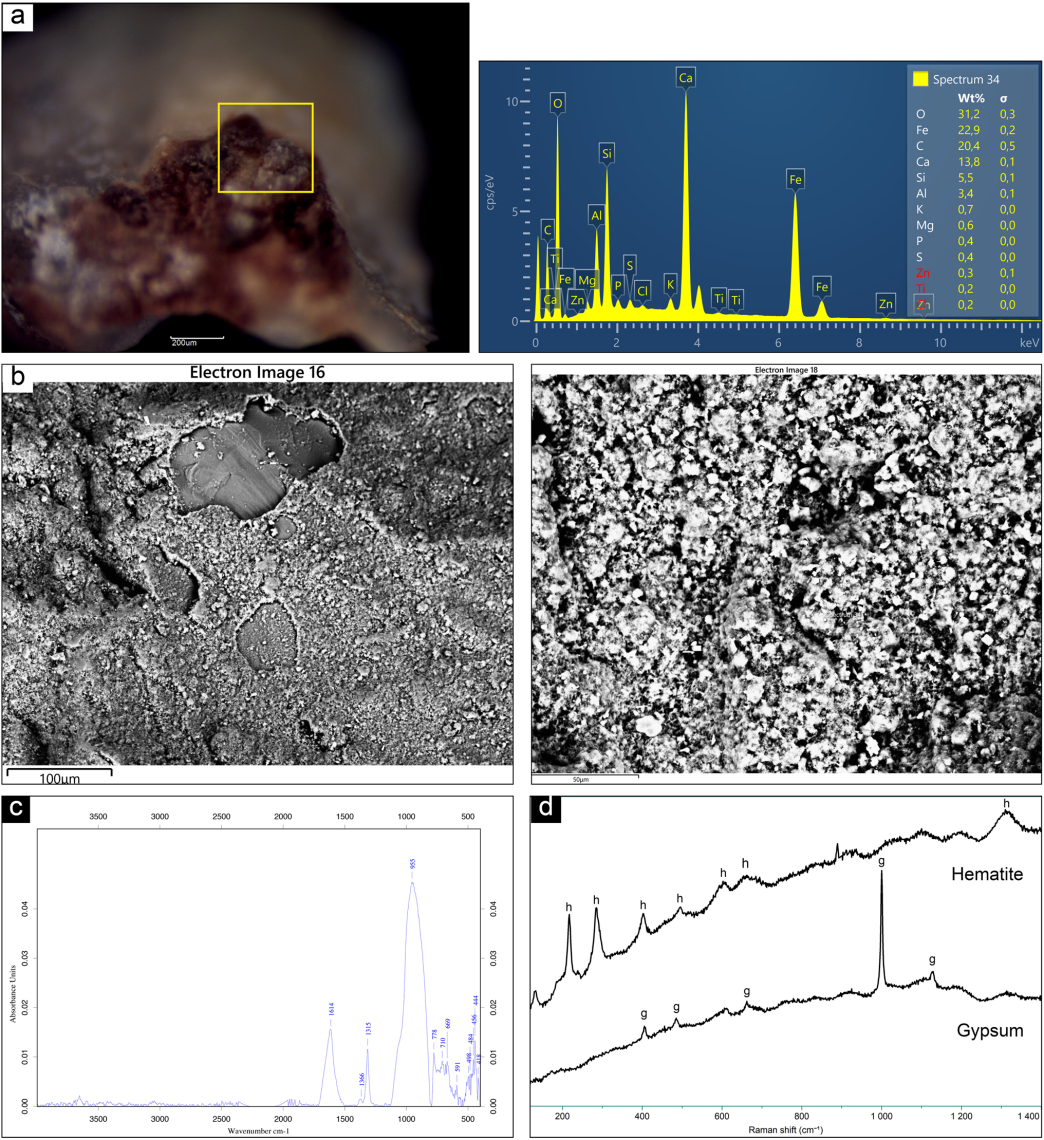
Summary of the analytical results obtained on sample ALI4: **a** Microscopic view and indication of investigation area; **b** SEM micrographs (BSE) of the area at different magnification and related EDS spectra; **c** ATR-FTIR spectrum showing whewellite and calcium phosphate peaks; **d** mineralogical phases detected by micro-Raman spectroscopy

The reddish area identified under the microscope (Fig. 6a) was observed and analyzed through SEM–EDS spectroscopy at different magnification, revealing a coating structure and a significative presence of Fe, Ca, and S, besides other elements attributable to the rocky substrate, such as Si, Al, Na, and Mg, (Fig. 6b). For this reason, a chemical mapping was carried out, in order to better understand their distribution and make hypothesis regarding the nature of the compounds present over the sample. This analysis showed different layers covering the bedrock. The main constituents of that film were Ca and Fe. Over it, however, was distinctively visible a formation characterized by a strong concentration of S. The presence of calcium and phosphorus showed by the SEM–EDS analyses can be correlated with FTIR findings, specifically with the presence of whewellite (1614 m, 1315 m, and 778 vs cm−1) (Bazin et al. 2016; Rampazzi et al. 2004) and calcium phosphate (955vs, 710w, 498w, 456w, 418w cm−1) (Grunenwald et al. 2014). For what concerns the mineralogical phase related to the application of pigment over the rock, hematite was detected by means of Raman spectroscopy (peaks at 216, 284, 403, 494, 602, 662, 1313 cm−1), suggesting the use of a natural red ochre (Fig. 6c and d). On the base of the morphological evidence and of the outcome of micro-Raman spectroscopic analyses, the presence of sulfur can be related to the deposition of gypsum, presenting Raman peaks at 406, 485, 609, 1000, 1127 cm^−1^ (Fig. 6d). This accretion represents a further layer of patina, deposited due to weathering phenomena. The contribution of the granitic substrate comprised the signals of quartz, detected by means of micro-Raman and ATR-FTIR, and feldspar detected by ATR-FTIR.

### ALI5

**Fig. 7:**
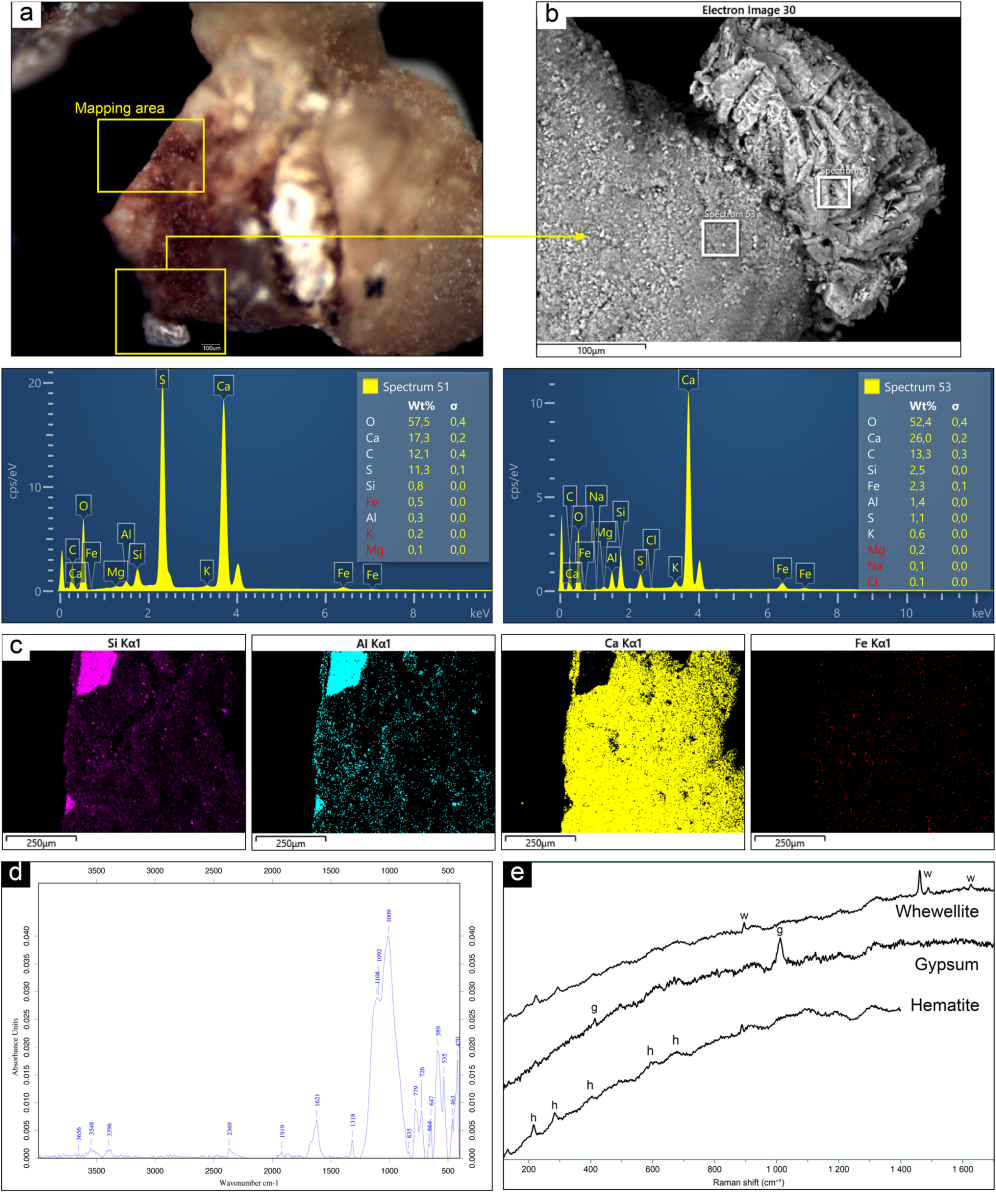
Summary of the analytical results obtained on sample ALI5: **a** microscopic view and indication of investigation areas; **b** SEM–EDS micrograph (BSE) on the pigment area and the gypsum crust and related EDS spectra; **c** SEM–EDS chemical mapping showing the distribution of Si, Al, Ca, and Fe and the presence of a calcic patina; **d** ATR-FTIR spectrum showing the peaks of whewellite and iron oxide; **e** mineralogical phases detected by micro-Raman spectroscopy

The microscopic visual inspection on sample 5 allowed to identify an area with a significative concentration of pigment (Fig. 7a). The same area was observed and analyzed by SEM–EDS technique. The backscattered mode and the further chemical analyses showed two very differentiate areas over the sample, also from the morphological point of view. Under SEM observation, the red area of the sample displayed a fine grain size, if compared with the adjacent crystalline formation. This latest was marked by the presence of S, while the former one, attributable to the painted surface, was characterized by the presence of Ca and Fe (Fig. 7b). Afterwards, the chemical mapping of the various element detected by EDS analyses over the painted areas highlighted the overlapping spatial distribution of Fe and Ca, thus suggesting the presence of a layer of calcic patina over the pigment. Moreover, the chemical mapping showed the neat distinction between the bedrock (Si, Al) and the overlaying compounds (Ca, Fe, S) (Fig. 7c). Micro-Raman analysis helped confirm this hypothesis, as it detected the presence of calcium oxalate, specifically whewellite (peaks at 894, 1461, 1488, 1626 cm^−1^) together with hematite (214, 283, 401, 601, and 659 cm^−1^) and gypsum (413, 1012 cm^−1^), this latest related to the crystalline formation enriched in S, observed by SEM and described above. FTIR results also reported the strong contribution of whewellite over this sample (peaks at 1313vs and 1610vs cm^−1^), the presence of iron oxide (599w cm^−1^), accordingly with the observations made by SEM–EDS and micro-Raman spectroscopy  (Fig. 7d and e), and the presence of feldspar, aluminosilicates, quartz, and carbonate.

### Substrate

**Fig. 8:**
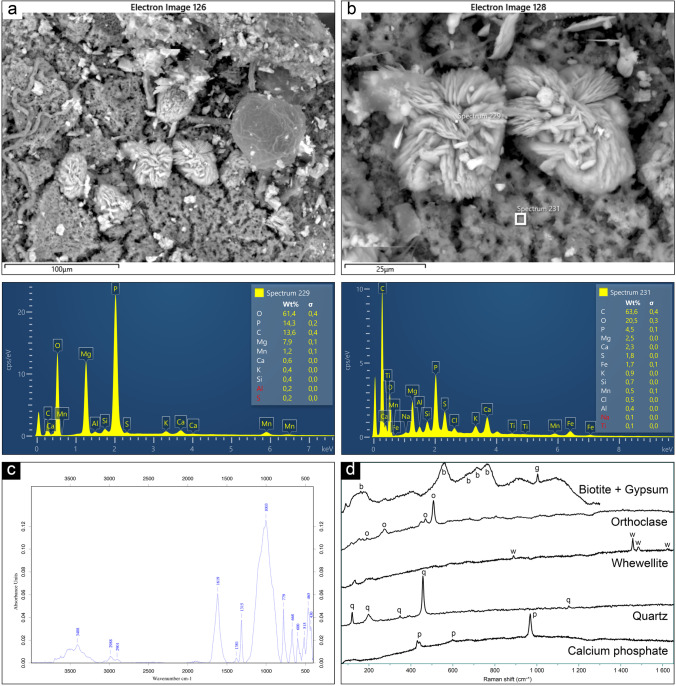
Summary of the analytical results obtained on substrate samples: **a** SEM micrograph (BSE) of bio-colonization structures; **b** detail of the phosphatic formations showed in Fig. 8a observed and analyzed by SEM–EDS, and related spectra; **c** ATR-FTIR spectrum showing the peaks of feldspar, quartz, and whewellite; **d** main mineralogical phases pertaining to the substrate and its patina, detected by micro-Raman spectroscopy

The main phases associated with the substrate (samples ALISU1 and ALISU2) were identified through the multi-technique approach. Through SEM–EDS analyses, some interesting features were shown, such as the presence of bio-colonization structures and phosphatic formations with a peculiar morphology on sample ALISU2 (Fig. 8a and b). ATR-FTIR detected feldspars (peaks at 1003vs, 668 m, 465 m, and 430 m cm^−1^), quartz (779 m cm^−1^), iron oxides, and other metal oxides (600w and 515w cm^−1^), which characterized the granitic bedrock. Consistently, µ-Raman spectroscopy detected quartz (119, 196, 255, 348, 384, 393, 456 s, 1150 cm^−1^), biotite (159, 558, 714, 759, 671 cm^−1^), and orthoclase (190, 274, 469, 506 cm^−1^). Moreover, whewellite was observed both through ATR-FTIR (peaks at 1619 s and 1315 cm^−1^) and micro-Raman (peaks at 888, 1457, 1484, 1623 cm^−1^). Gypsum was detected on substrate samples through µ-Raman spectroscopy (peaks at 1003 cm^−1^) together with bands attributable to calcium phosphate (peaks at 430, 441, 598, 968 cm^−1^) (Fig. 8c and d). Whewellite, gypsum, and calcium phosphate are attributable to the formation of patina, the same observed on pigment samples.

## Discussion

**Fig. 9:**
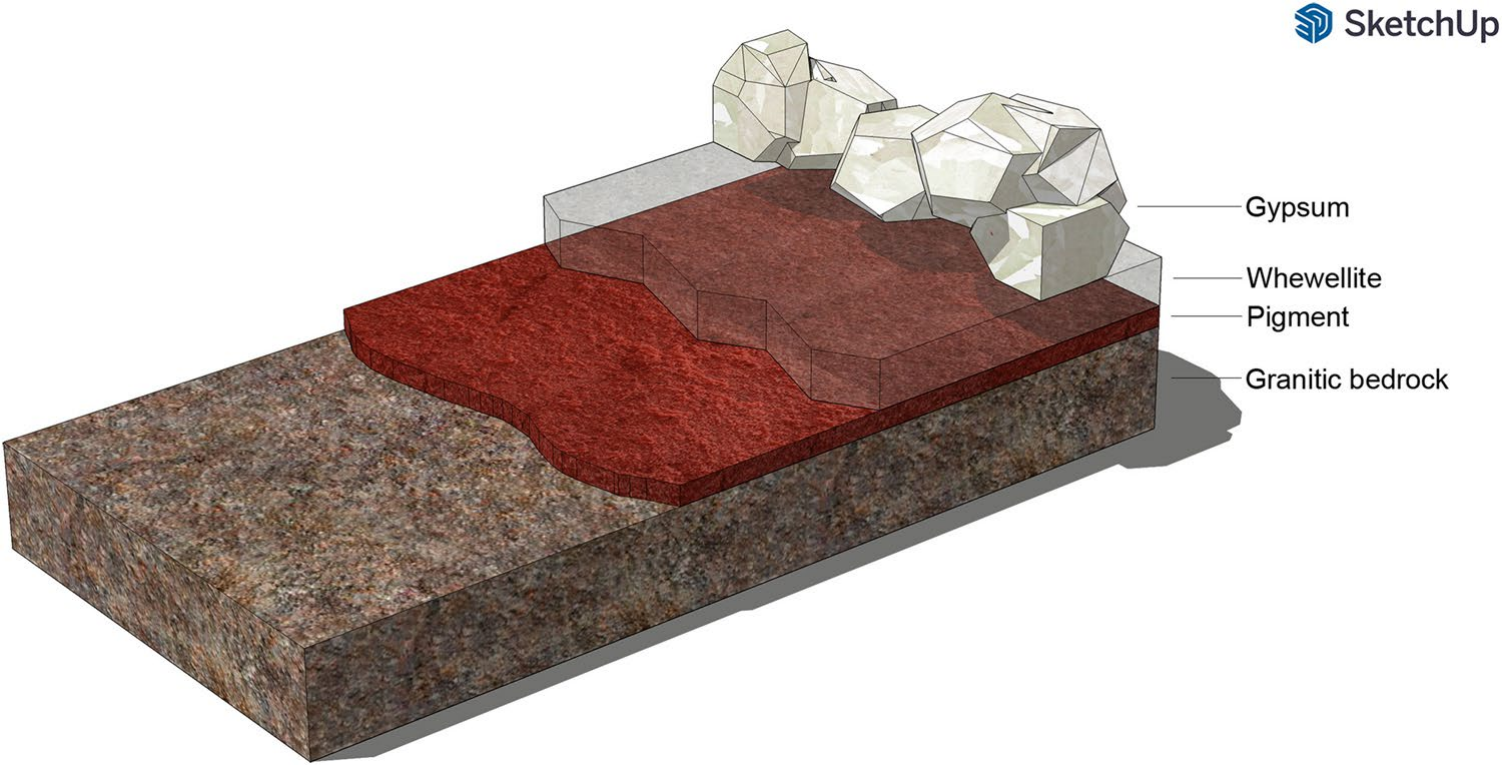
3D infographic scheme made using *SketchUp for Web* software. It shows the pattern of micro-stratigraphic sequence observed on pigment samples from “Abrigo del Lince” rock art shelter

Microscopic observation and geochemical characterization of pigment samples revealed a variegated, however quite homogeneous set of elements and compounds over the substrate. Those phases seemed to follow the same succession pattern, showing a clear distinction between the rocky substrate, the pigment layer, and the subsequent formation of patina. The main phases involved in this sequence are as follows: the granitic bedrock; the pigment layer, immediately over the rock and coming from an Fe-bearing material; a widespread and thin calcic encrustation layer; gypsum in the form of thick and well-developed crystal accretions (Fig. 9). The characterization of the substrate provided a term of comparison against which evaluate and interpret the data recorded during samples analysis, for example for the assessment of calcium contribution. Phosphatic structures observed on rock samples should be related to biological activities and/or presence of organic matter (Gallinaro and Zerboni 2021; Prinsloo 2007), which was indeed reported in field observations. Below is described and discussed the micro-stratigraphic sequence identified through the integration of microscopic observation and chemical analysis.

### Paint layer — discussion on pigment composition:

**Fig. 10:**
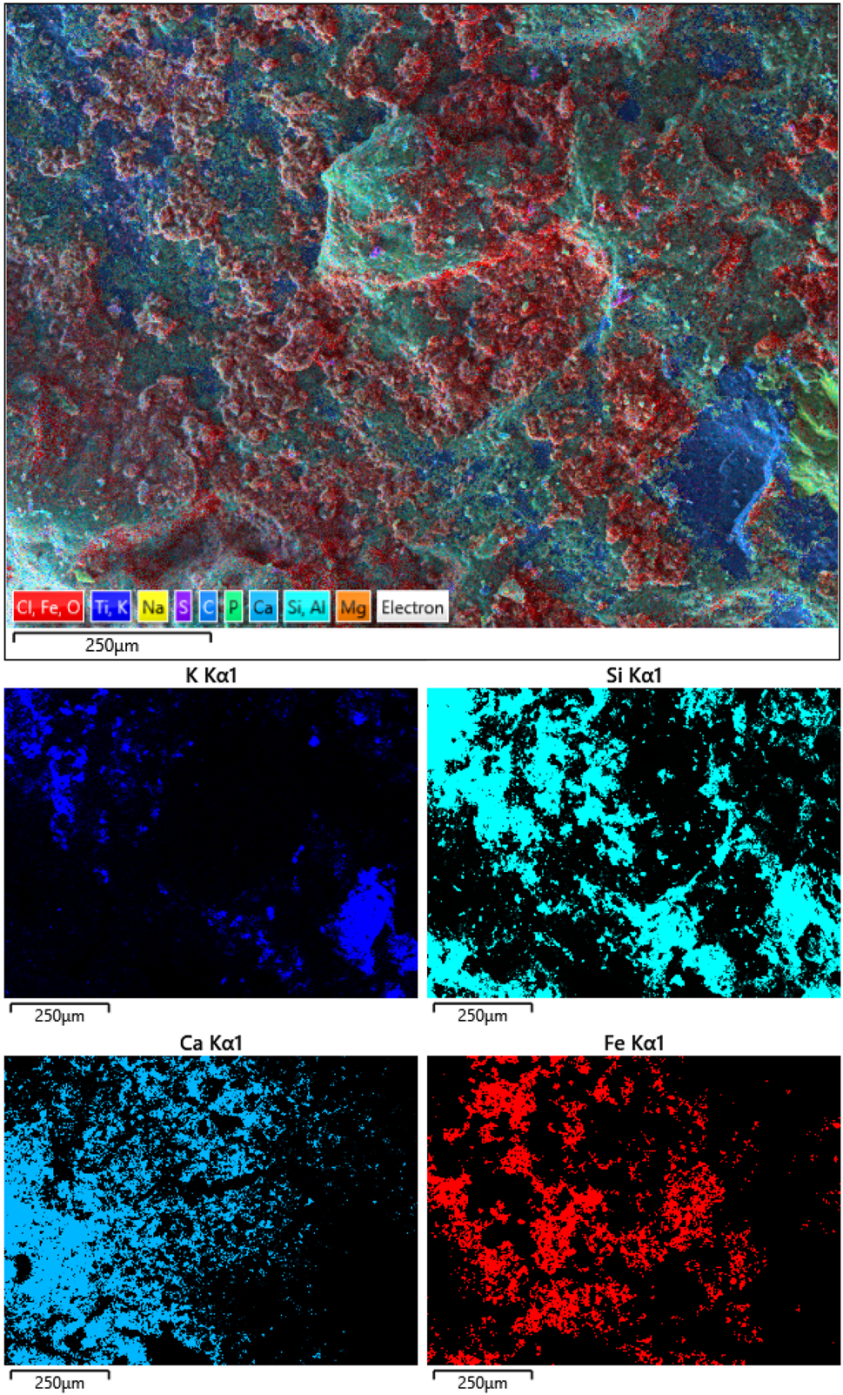
SEM–EDS chemical mapping over the pigment area on ALI2, showing the distribution of K, Si, Ca, and Fe and the areas with the main concentration of pigment. The widespread presence of a calcic patina is also remarkable

The outcome of the integrated analytical approach allowed to correlate this layer to the pictorial material applied on the rock, which conceive the pictographs. The pigment composition suggests the use of red ochre, as widely evidenced in several studies on prehistoric rock art, even conducted in the same geographical area (Iriarte et al. 2009; Rosina et al. 2019, 2018). The multimodal inspection of the layer containing hematite was performed comparing microscopic photographs and SEM–EDS chemical maps. This latest, carried out on sample ALI2, is representative of the distribution of Fe particles over the rocky panels (Fig. 10), which closely approximate the visual data from microscopic observation of pigment areas. On SEM–EDS elemental maps, however, the distribution could appear more discontinuous. In this regard, it must be pointed out that, despite the visibility of the reddish color of the pigment is due to the presence of iron oxides (Chalmin et al. 2017), those are not the only pigment constituents, but just the distinctive ones. Red ochers, in fact, contain also other minerals, such as silicates and aluminosilicates, and trace elements depending on their provenance and genesis (Velliky et al. 2019; MacDonald et al. 2011), while iron oxides such as hematite are considered the chromophores (Rousaki et al. 2018).

### Calcic layer — discussion on the role of patina in conservation of rock art pictographs

Calcium is one of the most abundant elements detected on samples, also in terms of frequency and distribution. This is, partially, due to the presence of gypsum concentrated in specific areas. However, remarkably, calcium appears to pertain mainly to a widespread and thin calcic encrustation layer, which covers the pigment without hiding it, at least from a visual point of view. The compositional map performed by SEM–EDS technique on sample ALI3 and ALI5 shows the clear distinction between the rocky substrate, rich in Al and Si, and the overlaying calcic coating (Figs. 5b and 7c). Underneath this layer and along its margins, the concentration of Fe particles related to the pigment is visible. Results of the chemical and mineralogical analysis of this compound suggest that whewellite and, in the case of ALI4, calcium phosphate is the main constituent of this first layer of patina over the pictographs. Those compounds are widely reported in the framework of geochemical studies on rock art, as well as of rock weathering studies (Gallinaro and Zerboni 2021). They could occur in relation to biomineralization processes, associated to the presence of biofilms or organic matter, over the rock surface, which are present also at *Abrigo del Lince* rock art shelter. Moreover, Gallinaro and Zerboni (2021) reported that calcium oxalates can be occasionally associated with gypsum, as observed in the present study for ALI5. Whewellite is considered to be a common constituent of the uppermost crust layer of bedrocks, which forms naturally due to weathering phenomena. Although the role of biofilms in rock art conservation is still debated, the presence of oxalates, possibly dependent to organisms’ metabolic processes, should not necessarily be considered an issue for the conservation of pictographs. For what concerns the consolidation of porous limestone, for example, calcium oxalate is the desired end product when ammonium oxalate treatment is applied to calcareous stone, due to its greater resistance to deterioration (Dreyfuss 2020). Similarly, especially if even and thin, the layer of oxalates could prevent the rock art pigment to decay and thus improve its stability (Gallinaro and Zerboni 2021; Rampazzi 2019).

### Gypsum

**Fig. 11:**
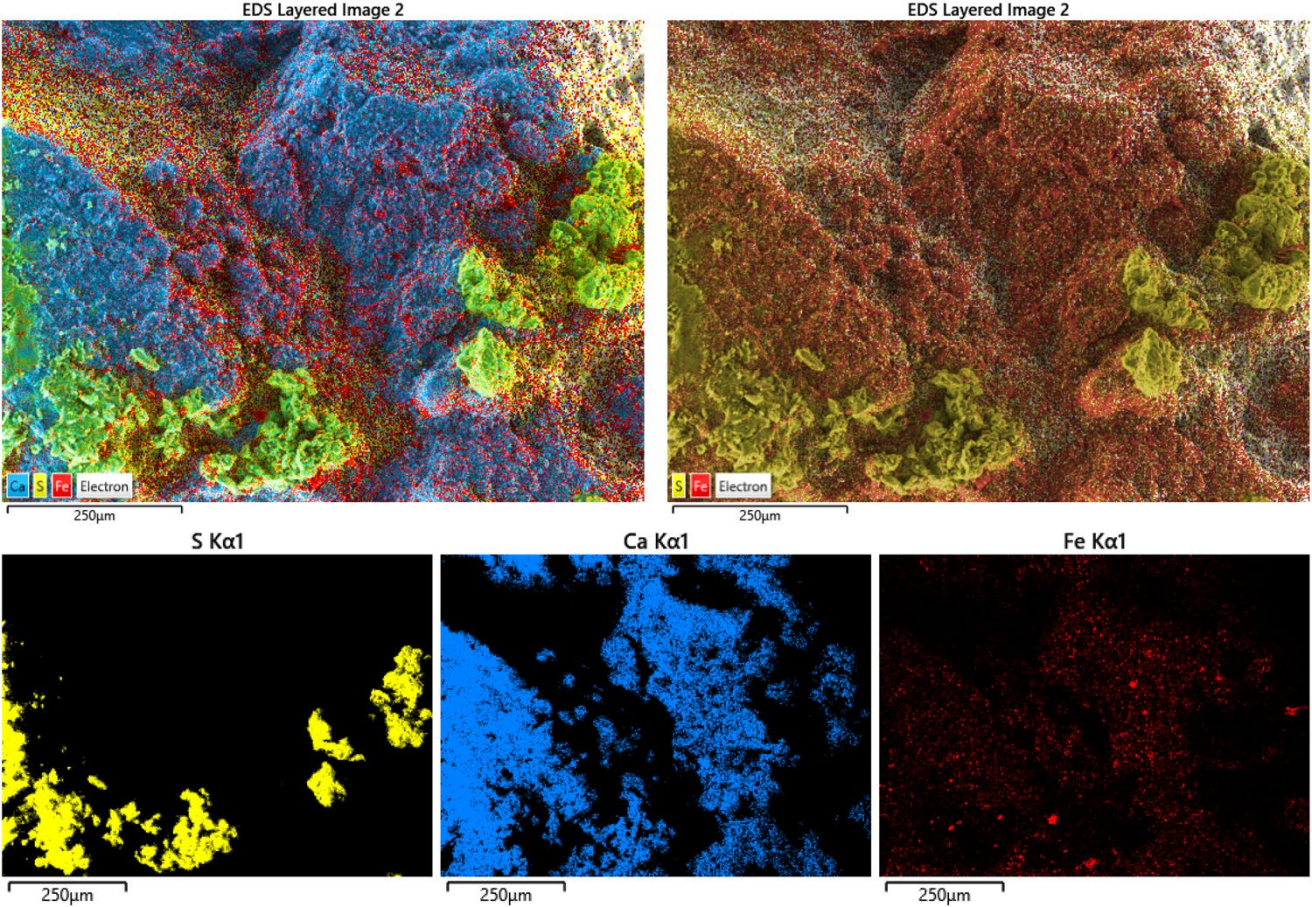
SEM–EDS chemical mapping, displayed with and without calcium contribution, performed over the pigment area of sample ALI4. Miniatures show the distribution of S, Ca, and Fe. The lower visibility of this latest element is attributable to the formation of a calcic encrustation and of a subsequent sulfate accretion, attributable to gypsum, over the pigment

The second phase constituting the patina is a discontinuous layer of gypsum, as found also by Gallinaro and Zerboni (2021). SEM–EDS elemental maps clearly show the morphology and the spatial distribution of this thick accretion (Fig. 11). This mineralogical phase tends to cover all the other layers, often hiding them, and its presence was not detected under the pigment or the oxalate layers (Figs. 10 and 7). For this reason, it could be excluded that gypsum was used during pigment or rock panel preparation, pointing to an atmospheric source of the sulfate. Its presence is attributable to the weathering processes affecting the rock panels, such as exposure to rainwater and water runoff which favour the precipitation of soluble salts (Chalmin et al. 2017). On historical stone monuments, gypsum is found as the main constituent of the so-called black crusts (Comite et al. 2021; Pozo-Antonio et al. 2017). Those formations firmly adhere to the stone surface and, due to their texture, they accumulate materials from the atmosphere (Pozo-Antonio et al. 2021). Even if their formation processes remain not totally understood, gypsum black crusts are considered an important alteration and deterioration factor in cultural heritage studies, for several reason. They hide and alter the legibility of the artworks they lay on. Moreover, due to their structure, they embed compounds from the environment which are potentially damaging, or which can favour the establishment of secondary alterations (Comite et al. 2021). More importantly, the different physic and mechanical behavior of the uppermost gypsum crust against the granitic substrate can induce detachments and the loss of surface material (Pozo-Antonio et al. 2019). For these reasons, the presence of gypsum on rock art paintings, even if at a lower extent, should be considered a potential risk for the preservation and fruition of pictographs and rock art panels.

## Conclusion

The present study contributed to the acquisition of morphological, chemical, and mineralogical information about pigment and alteration phases detected in rock art figures painted over a granitic substrate in the open-air archeological site of “Abrigo del Lince,” in the province of Badajoz (Extremadura, Spain). In order to collect a comprehensive data set and formulate a consistent interpretation, the multi-technique approach was fundamental. Microscopic observation, SEM–EDS analysis, micro-Raman spectroscopy, and ATR-FTIR were integrated to obtain a multispectral overview of the samples, focusing on selected areas. This method of investigation allowed to highlight the varied composition of samples, to characterize their components and, finally, to describe the alteration pattern which connects them. Hematite seems to be the main constituent of the pigment, while whewellite and gypsum appeared to be the main constituent of the patina layer, deposited over the figures due to weathering and bio-mineralization processes. Despite the presence of a layer of patina, the legibility, fruition, and conservation of rock art evidence at *Abrigo del Lince* rock art site should not be necessarily considered at risk at the moment. However, it is good to emphasize the importance of monitoring the growth of biofilm and patina accretion, especially for what concern gypsum encrustation.

## Data Availability

The data generated and analyzed during this study are included in this article. Other datasets used during the current study are available from the corresponding author on reasonable request.

## References

[CR1] Acosta Martínez P (1968). La pintura rupestre esquemática en España.

[CR2] Breuil H (1933) Les peintures rupestres schématiques de la Péninsule Ibérique, Foundatión Singer-Polignac, IV volúmenes, Lagny

[CR3] Bazin D, Jouanneau C, Bertazzo S, Sandt C, Dessombz A, Réfrégiers M, Dumas P, Frederick J, Haymanng J, Letavernier E, Ronco P, Daudon M (2016). Combining field effect scanning electron microscopy, deep UV fluorescence, Raman, classical and synchrotron radiation Fourier transform Infra-Red Spectroscopy in the study of crystal-containing kidney biopsies. C R Chim.

[CR4] Chalmin É, Castets G, Delannoy JJ, David B, Barker B, Lamb L, ..., Gunn R (2017) Geochemical analysis of the painted panels at the “Genyornis” rock art site, Arnhem Land, Australia. Quat Int 430 Part A:60–80. 10.1016/j.quaint.2016.04.003

[CR5] Collado Giraldo H (2007) Arte rupestre prehistórico en Extremadura: 1997–2006. In: R. Balbín Behrmann (Ed.), Arte Prehistórico al aire libre en el Sur de Europa, pp. 287–490

[CR6] Collado Giraldo H (2009) Propuesta para la clasificación funcional y cronológica del arte rupestre esquemático a partir del modelo extremeño. In: Cruz-Auñon Briones R, Ferrer Albelda E (Eds.), Estudios de Prehistoria y Arqueología en homenaje a Pilar Acosta Martínez, pp. 89–107

[CR7] Collado Giraldo H, Garcia JJ (eds.) (2005) Arte rupestre en el Parque Natural de Monfragüe: el sector oriental. Corpus de Arte Rupestre en Extremadura, vol. I. Consejería de Cultura, Mérida

[CR8] Collado Giraldo H, García Arranz JJ (2009). Pintura rupestre esquemática sobre granito en la provincia de Cáceres: los ejemplos de la Cueva Larga del Pradillo y Los. ZEPHYRUS Revista De Prehistoria y Arqueología.

[CR9] Collado Giraldo H, García JJ (edits.) (2017): Arte Rupestre en la cornisa de La Calderita (término municipal de La Zarza). Corpus de Arte Rupestre en Extremadura, vol. IV, Consejería de Cultura e Igualdad, Mérida

[CR10] Collado Giraldo H, García JJ, Domínguez I, Rivera E, Nobre LF (2009) “Novedades en el arte postpaleolítico de Extremadura”. Actas del IV Congreso “El arte rupestre del arco mediterráneo de la Península Ibérica”, pp. 123–130

[CR11] Collado Giraldo H, Rosina P, García Arranz JJ, Gomes H, Nobre L, Domínguez García I, Duque Espino D, Fernánde Valdés J, Blasco Laffón E, Torrado Cárdeno JM, Rodríguez Dorado L, Rivera Rubio E, Nacarino de los Santos M, Capilla Nicolás J, Pérez Romero S (2014) El arte rupestre esquemático del Arroyo Barbaón (Parque Nacional de Monfragüe, Cáceres): contextualización arqueológica y caracterización de pigmentos. ZEPHYRUS. Revista de Prehistoria y Arqueología LXXIV:15–39. 10.14201/zephyrus2014741539

[CR12] Comite V, Miani A, Ricca M, La Russa M, Pulimeno M, Fermo P (2021). The impact of atmospheric pollution on outdoor cultural heritage: an analytic methodology for the characterization of the carbonaceous fraction in black crusts present on stone surfaces. Environ Res.

[CR13] Domínguez García IM, Collado Giraldo H, García Arranz JJ (2010) Evolución de la metodología de la investigación aplicada a la pintura rupestre esquemática en la provincia de Badajoz (Extremadura, España). Anais Do Congresso Internacional de Arte Rupestre - IFRAO. Parque Nacional Da Serra Da Capivara, Piauí, Brasil 381–393

[CR14] Domínguez García IM, Collado Giraldo H, García Arranz JJ (2013) Un siglo de investigación para la pintura rupestre esquemática de la provincia de Badajoz. Evolución de la metodología y nuevas aportaciones. In: Martínez García J, Hernández Pérez MS (Eds.), Actas del II Congreso de arte rupestre esquemático en la Península Ibérica. Ayuntamiento de Vélez-Blanco 279–286

[CR15] Dreyfuss T (2020). Artificially induced calcium oxalate on limestone in urban environments–new findings. J Cult Herit.

[CR16] EN 16085:2012- Conservation of cultural property - Methodology for sampling from materials of cultural property – General rules

[CR17] Gallinaro M, Zerboni A (2021). Rock, pigments, and weathering. A preliminary assessment of the challenges and potential of physical and biochemical studies on rock art from southern Ethiopia. Quatern Int.

[CR18] Gheco L, Tascon M, Etcheberry EA, Quesada M, Marte F (2020). Looking for paint mixtures to glimpse pictorial techniques: a micro-stratigraphic physicochemical approach to the rock art from the Oyola’s Caves (Argentina). Heritage Science.

[CR19] Green H, Gleadow A, Finch D, Hergt J, Ouzman S (2017). Mineral deposition systems at rock art sites, Kimberley, Northern Australia—field observations. J Archaeol Sci Rep.

[CR20] Grunenwald A, Keyser C, Sautereau AM, Crubézy E, Ludes B, Drouet C (2014). Revisiting carbonate quantification in apatite (bio) minerals: a validated FTIR methodology. J Archaeol Sci.

[CR21] Iriarte E, Foyo A, Sánchez MA, Tomillo C, Setién J (2009). The origin and geochemical characterization of red ochres from the Tito Bustillo and Monte Castillo caves (northern Spain). Archaeometry.

[CR22] Kurniawan R, Kadja GTM, Setiawan P, Burhan B, Oktaviana AA, Hakim B, Aubert M, Brumm A, Ismunandar,  (2019). Chemistry of prehistoric rock art pigments from the Indonesian island of Sulawesi. Microchem J.

[CR23] MacDonald BL, Hancock RGV, Cannon A, Pidruczny A (2011). Geochemical characterization of ochre from central coastal British Columbia Canada. J Archaeol Sci.

[CR24] Pozo-Antonio JS, Pereira MFC, Rocha CSA (2017). Microscopic characterisation of black crusts on different substrates. Sci Total Environ.

[CR25] Pozo-Antonio JS, Papanikolaou A, Philippidis A, Melessanaki K, Rivas T, Pouli P (2019). Cleaning of gypsum-rich black crusts on granite using a dual wavelength Q-Switched Nd: YAG laser. Constr Build Mater.

[CR26] Pozo-Antonio JS, Alonso-Villar EM, Rivas T (2021). Efficacy of mechanical procedures for removal of a lichen and a gypsum black crust from granite. J Build Eng.

[CR27] Prinsloo LC (2007). Rock hyraces: a cause of San rock art deterioration?. J Raman Spectrosc.

[CR28] Rampazzi L, Andreotti A, Bonaduce I, Colombini MP, Colombo C, Toniolo L (2004). Analytical investigation of calcium oxalate films on marble monuments. Talanta.

[CR29] Rampazzi L (2019). Calcium oxalate films on works of art: a review. J Cult Herit.

[CR30] Rousaki A, Vargas E, Vázquez C, Aldazábal V, Bellelli C, Calatayud MC, Hajduk A, Palacios O, Moens L, Vandenabeele P (2018). On-field Raman spectroscopy of Patagonian prehistoric rock art: pigments, alteration products and substrata. TrAC, Trends Anal Chem.

[CR31] Rosina P, Gomes H, Collado H, Nicoli M, Volpe L, Vaccaro C (2018). Μicro-Raman spectroscopy for the characterization of rock-art pigments from Abrigo del Águila (Badajoz – Spain). Opt Laser Technol.

[CR32] Rosina P, Collado H, Garcês S, Gomes H, Eftekhari N, Nicoli M, Vaccaro C (2019). Benquerencia (La Serena - Spain) rock art: an integrated spectroscopy analysis with FTIR and Raman. Heliyon.

[CR33] Ruiz JF, Hernanz A, Armitage RA, Rowe MW, Viñas R, Gavira-Vallejo JM, Rubio A (2012). Calcium oxalate AMS 14C dating and chronology of post-Palaeolithic rock paintings in the Iberian Peninsula. Two dates from Abrigo de los Oculados (Henarejos, Cuenca, Spain). J Archaeol Sci.

[CR34] Sanjurjo-Sánchez J, Romaní JRV, Alves C (2012). Comparative analysis of coatings on granitic substrates from urban and natural settings (NW Spain). Geomorphology.

[CR35] Sepúlveda M (2021). Making visible the invisible. A microarchaeology approach and an archaeology of color perspective for rock art paintings from the southern cone of South America. Quatern Int.

[CR36] Velliky EC, Barbieri A, Porr M, Conard NJ, MacDonald BL (2019). A preliminary study on ochre sources in Southwestern Germany and its potential for ochre provenance during the Upper Paleolithic. J Archaeol Sci Rep.

